# Elafin, an inhibitor of elastase, is a prognostic indicator in breast cancer

**DOI:** 10.1186/bcr3374

**Published:** 2013-01-15

**Authors:** Kelly K Hunt, Hannah Wingate, Tomoya Yokota, Yanna Liu, Gordon B Mills, Fan Zhang, Bingliang Fang, Chun-Hui Su, Ming Zhang, Min Yi, Khandan Keyomarsi

**Affiliations:** 1 Department of Surgical Oncology, The University of Texas MD Anderson Cancer Center, 1400 Pressler Street, Unit 1484, Houston, TX 77030, USA; 2 Department Experimental Radiation Oncology, The University of Texas MD Anderson Cancer Center, 1515 Holcombe Boulevard, Unit 066, Houston, TX 77030, USA; 3 Department of Systems Biology, The University of Texas MD Anderson Cancer Center, 7455 Fannin Street, Unit 950, Houston, TX 77054-1901, USA; 4 Department Thoracic and Cardiovascular Surgery, The University of Texas MD Anderson Cancer Center, 1400 Hermann Pressler Drive, Unit 1489, Houston, TX 77030, USA; 5 Division of Gastrointestinal Oncology, Shizuoka Cancer Center, 1007 Shimonagakubo, Nagaizumi-cho, Sunto-gun, Shizuoka 411-8777 Japan; 6 Department of Molecular Pharmacology and Biological Chemistry, Feinberg School of Medicine, Northwestern University, 303 E. Superior Street, Chicago, IL 60611, USA

## Abstract

**Introduction:**

Elafin is an elastase-specific inhibitor with increased transcription in normal mammary epithelial cells compared to mammary carcinoma cells. In this report, we test the hypothesis that inhibition of elastase, through induction of elafin, leads to inhibition of human breast cancer cell viability and, therefore, predicts survival in breast cancer patients.

**Methods:**

Panels of normal and immortalized breast epithelial cells, along with breast carcinoma cells, were used to examine the impact of adenoviral-mediated elafin expression or shRNA-mediated inhibition of elastase on the growth of cells and xenografts in nude mice. To determine the prognostic significance of decreased elafin in patients with invasive breast cancer, previously published gene array datasets were interrogated.

**Results:**

Elafin expression had no effect on non-tumorigenic cells but resulted in marked inhibition of cell growth in breast cancer cell lines. Control-treated xenografts generated a tumor burden that necessitated sacrifice within one month of initial treatment, whereas xenograft-bearing mice treated with Ad-Elafin were alive at eight months with marked reduction in tumor growth. Elastase inhibition mimicked these results, showing decreased tumor cell growth *in vitro *and *in vivo*. Low expression of elafin gene correlated with significantly reduced time to relapse, and when combined with high expression of elastase gene was associated with decreased survival in breast cancer patients.

**Conclusion:**

Our data suggest that elafin plays a direct role in the suppression of tumors through inhibition of elastase and thus serves as a prognostic indicator for breast cancer patients.

## Introduction

Polymorphonuclear leukocyte elastase (hereafter referred to as elastase) disintegrates matrix proteins [1], implicating this enzyme in breast cancer cell invasion and metastasis. Elastase is produced by neutrophils and also by human breast cancer cells but not by normal breast epithelial cells in culture [2]. Increased levels of elastase have been shown to be strongly associated with recurrence and death in breast cancer patients [3]. A study of 313 breast cancer patients with a median of 18.5 years of follow-up showed that elastase in tumor extracts was an independent prognostic factor associated with increased risk of recurrence [4]. These studies suggest that elastase could have a role in tumor progression leading to metastasis in breast cancer. The use of elastase inhibitors to reverse the effects of elastase in acute lung injury and to inhibit formation of atherosclerotic plaques has been explored in experimental models [5,6]. A natural inhibitor of elastase, called elafin, was identified by subtractive hybridization comparing genes expressed in normal human mammary epithelial and human breast carcinomas [7]. Zani *et al. *showed that elafin is a potent inhibitor of elastase activity *in vitro *[8]. Adenoviral delivery of elafin was able to protect endothelial cells from elastase-induced production of cytotoxic products, which resulted in a decrease of atherogenic stimuli and inhibition of elastase-induced lung hemorrhage [5,6]. Lastly, in a mouse model of colitis, elafin overexpression inhibited elastase-associated inflammation [9]. These studies suggest that elafin inhibits the function of elastase *in vivo*.

A lack of elastase inhibition would provide a significant advantage to cancer cells with respect to the metastatic process. Elafin is expressed in well-differentiated squamous cell carcinoma of the skin and esophagus but is lost in poorly differentiated tumors [10-13]. Elafin was found in tumor cell nests, and DNA fragmentation was noted in these cell layers, suggesting that elafin was involved in induction of apoptosis [13]. Elafin was found in the cytoplasm just beneath the cell membrane, and elastase was present adjacent to these elafin-positive cells [11,12], suggesting that elafin is involved in suppressing the progression of tumors, possibly through inhibition of elastase.

However, it is unclear what the relationship between elafin and elastase is in cells and whether elafin can inhibit elastase mediated tumor progression. We investigated the role of elafin expression and inhibition of elastase in mediating tumor-specific growth inhibition in breast cancer cells and the prognostic significance of elafin in predicting outcomes in breast cancer patients.

## Materials and methods

### Microarray analysis

Gene expression and patient outcomes data were obtained from previously published datasets [14,15]. Affymetrix Human U133a Gene chips (Santa Clara, CA, USA) were used to assess the expression of 22,000 transcripts in each cohort. The Wang dataset was from analysis of total RNA obtained from frozen tumor samples from 286 patients with lymph node-negative breast cancer who had not received systemic adjuvant therapy [15]. The expression data for elafin (*PI3*) and elastase (*ELA2/ELANE*) genes and the relationship between their expression and time to relapse were analyzed using a log-rank test and shown using Kaplan-Meier survival plots. The cutoffs for high versus low expression were optimized to achieve the lowest *P-*value. The ranges of expression using the *PI3 *probes41469_at and 203691_at were 4.73 to 8.59 and 5.02 to 10.23, respectively, and the cutoffs were optimized at 5.042 and 5.44, respectively. The estrogen receptor (ER) status was available for each tumor sample, and the elafin levels were compared between the ER-positive and ER-negative groups using the two-sample Student's *t*-test.

### Cell culture

Immortalized mammary epithelial cell lines 76NE6, 76NF2V, 76NY54H, 76NE7 and MCF-10A were gifts of Dr. Vimla Band (University of Nebraska Medical Center, Omaha, NE, USA) [16-18]. 76NE6 and 76NE7 were immortalized through transfection of normal mammary epithelial cells (76N) with the E6 and E7 genes of the HPV genome, rendering them p53 or pRb defective, respectively. 76NF2V and 76NY54H cells were also immortalized with the E6 gene. However, point mutations were introduced into the E6 gene so that these cells maintain functional p53 while being immortalized. MCF-10A cells were immortalized through long-term culture in serum free media. ER-positive breast carcinoma cell lines (MCF-7, ZR75T and BT20T), ER-negative breast carcinoma cell lines (T47D, HBL100, MDA-MB-436, MDA-MB-231 and MDA-MB-157) and NIH3T3 fibroblasts were obtained from the American Type Culture Collection (Manassas, VA, USA). Cells were cultured in medium from HyClone (Logan, UT, USA) containing serum obtained from Atlanta Biologicals, Inc. (Lawrenceville, GA, USA). The cells were cultured at 37°C in 6.5% CO_2_. All cells were authenticated by cytogenetic testing at the Characterized Cell Line Core Facility at MD Anderson Cancer Center and were verified as being free of mycoplasma contamination by PCR.

### Adenoviral vectors

Recombinant adenoviral vectors Ad-Luc and Ad-Elafin were constructed by inserting the cytomegalovirus promoter plus the transgene (luciferase or elafin) into the E1 region of an E1/E3-deleted type 5-adenovirus as described previously [9,19,20]. Virus titers were determined by measuring optical absorbance at A_260 _and by plaque-forming assays. Particle to plaque ratios fell between 10:1 and 100:1. All of the viral preparations were free of E1A contamination and endotoxins. Transduction efficiency was previously determined using an adenoviral vector containing the β-galactosidase reporter gene under control of the cytomegalovirus promoter. In previous reports, we showed that at a multiplicity of infection of 2,000 viral particles (vp), 85% to 95% of the cells were infected, and the recombinant adenoviruses induced high levels of transgene expression [21].

### Western blot analysis

Total cell extracts were prepared with cell lysate buffer, and cell extracts were analyzed for protein content by SDS-PAGE as described previously [21]. Briefly, 50 μg of protein was subjected to electrophoresis on a 10% or 13% SDS-PAGE gel. Protein was then transferred to Immobilon P membranes (Millipore, Billerica, MA, USA), which were blocked overnight in BLOTTO (5% nonfat dried milk in 20 mM Tris, 137 mM NaCl and 0.05% Tween (pH 7.6)). After washing, the blots were incubated in primary antibodies for 2.5 h. Primary antibodies used were elafin (HM2063; HyCult Biotech, Uden The Netherlands) and actin (Chemicon International, Inc., Temecula, CA, USA). Blots were then incubated with horseradish peroxidase conjugated secondary antibodies at a 3:5,000 dilution in BLOTTO for 1 h, washed, and developed by chemiluminescence (Western Lightning Chemiluminescence Reagent Plus; Perkin-Elmer Life Sciences, Cambridge, UK) according to the manufacturer's instructions. Actin was used to standardize equal loading. Uncropped blots are shown in Additional file 1.

### Confocal microscopy

Cells (4 × 10^5^) were grown on poly-L-lysine-coated coverslips (Corning Biosciences, Lowell, MA, USA) in six-well plates for 12 h. Cells were fixed with 2% paraformaldehyde and incubated for 15 minutes with 70% ethanol, washed and covered with 1% gelatin. Cells were rinsed with PBS, permeabilized with 0.2% Triton X-100, blocked with 1% goat serum (Santa Cruz Biotechnology, Inc., Santa Cruz, CA, USA; sc-2043) and then incubated with antibody to either elafin (Santa Cruz Biotechnology; sc-20637) or elastase (Merck KGaA, Darmstadt, Germany; 481001) diluted 1:200 in 3% bovine serum albumin in a humidified box overnight at 4°C. Detection was performed with anti-rabbit Rhodamine Red-X-conjugated secondary antibodies (Invitrogen, Carlsbad, CA, USA; R6394; 1:400 dilution), or Alexa Fluor 555 or Alexa Fluor 488 goat anti-mouse antibodies (Invitrogen; A21428 and A11008 respectively; 1:300 dilution). For elastase shRNA experiments, two secondary antibodies were used to confirm knocked down expression as no antibody is available for Western blotting. Cells were rinsed, followed by the addition of one drop of mounting medium (Invitrogen; P36935) and 4',6-diamidino-2-phenylindole (DAPI). Imaging was performed on an Olympus FV500 confocal microscope (Olympus America Corporate Headquarters, Center Valley, PA, USA).

### Proliferation and invasion assays

For proliferation analyses, cells were seeded at 5 × 10^3 ^cells per well in 24-well plates, and cells were infected with Ad-Elafin (2,000 vp/mL and 2,500 vp/mL) or with Ad-Luc (2,000 vp/mL and 2,500 vp/mL) or mock-infected with PBS and evaluated by direct cell counting by hemocytometer of duplicate plates at Days 1, 2, 3 and 4. Invasion assays were carried out using Oris Cell Migration Assay Kit according to the manufacturer's instructions (Platypus Technologies, Madison, WI, USA). A total of 1 × 10^5 ^cells were seeded around stoppers that created a detection zone, and incubated overnight. The stoppers were removed from test wells but left in place in the pre-migration reference wells until assay readout. All wells received CellTracker Green (Platypus Technologies) to fluorescently stain the cells. Cell migration was measured by fluorescence signals in the detection zones using a plate reader. Fluorescence was monitored at excitation and emission wavelengths of 492 nm and 530 nm, respectively. Images of pre-migration wells (time = 0 h) and post-migration wells (time = 48 h) were acquired using fluorescence microscopy with an Olympus FV500 confocal microscope.

### shRNA-mediated down-regulation of elastase and elafin

shRNA vectors against elastase (TG310440) and a control vector containing a scrambled transcript (TR30012) were obtained from Origene (Rockville, MD, USA). Cells were transfected with 5 μg of vector using Genejuice reagent (Novagen, Madison, WI, USA) according to the manufacturer's instructions. Cells expressing these vectors were selected in α-minimal essential medium containing 2 μg/mL puromycin (Sigma, St. Louis, MO, USA) for four weeks. Single-cell clones were selected and expanded in culture medium supplemented with 0.1 mg/mL G418 and 2 μg/mL puromycin (Sigma) and screened by Western blot. Elastase activity was measured using MeOSuc-Ala-Ala-Pro-Val-pNA (Sigma-Aldrich) as a substrate. Lysates from 76NE6 cells with or without knock-down of elafin were incubated with 350 μg of 2 mM substrate for 48 hours in reaction buffer (0.1 M Tris 7.5, 0.5 M NaCl) and absorbance was measured at 405 nM.

### Mouse xenograft studies

Mice were housed five per cage in sterilized micro-isolator cages (Lab Products, Seaford, DE, USA) furnished with corncob bedding. Mice received care in accordance with the Animal Welfare Act, the National Institutes of Health "Guide for the Care and Use of Laboratory Animals" and the institutional guidelines of MD Anderson Cancer Center. All experiments were approved by the Institutional Animal Care and Use Committee at MD Anderson Cancer Center. A total of 1 × 10^6 ^cells were injected into the mammary fat pad of four- to six-week-old female Balb/c Nu/nu mice (Taconic, Germantown, NY, USA). For treatment with elafin, MDA-MB-468 breast cancer cells were xenografted. When the tumor size reached 100 mm^3^, mice were divided into treatment groups. The tumors were treated with 2 × 10^10 ^vp/mL Ad-Elafin (*n *= 6), 2 × 10^10 ^vp/mL Ad-Luc (*n *= 4), or PBS (*n *= 4) on Days 1, 5, 8 and 12. To observe effects of elastase shRNA on tumor growth, nude mice were injected with MDA-MB-231 breast cancer cells treated with a combination of either the two control vectors (*n *= 8) or the two elastase shRNA constructs (*n *= 8) in the mammary fat pads. The tumor volume was calculated every other day. Mice were euthanized when tumors were greater than 1.5 cm in diameter at the widest dimension of the tumor.

### Immunohistochemical analysis

Hematoxylin and eosin staining was performed on sections cut from tumor tissue embedded in paraffin blocks. The sections were stained with polyclonal antibodies to either elafin (Santa Cruz Biotechnology; sc-20637) or elastase (Calbiochem, Billerica, MA, USA; 481001) diluted 1:200 in 3% bovine serum albumin. Protein expression was visualized with avidin-biotin-peroxidase reagent using a Vectastain ABC kit (Vector Laboratories, Burlingame, CA, USA) according to the manufacturer's recommendations.

## Results

### Elastase inhibition decreases proliferation of breast cancer cells

**Figure 1:**
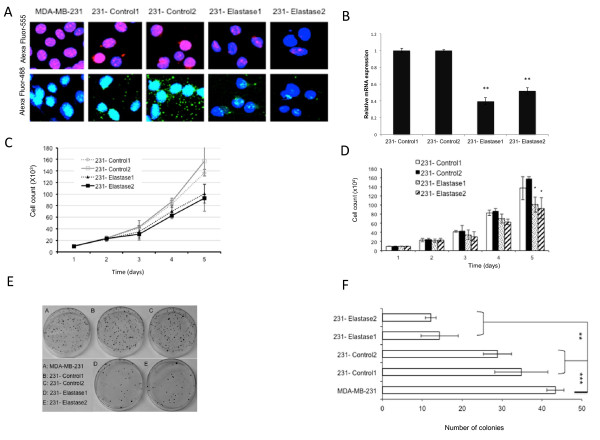
**shRNA-mediated downregulation of elastase decreases proliferation of MDA-MB-231 cells**. MDA-MB-231 cells were selected that stably expressed control, scrambled shRNA (231-Control1 and 231-Control2) or elastase-specific shRNA (231-Elastase1 and 231-Elastase2). **A**) Down-regulation of elastase was confirmed by confocal microscopy of each clone and the parental cells using Alexa Fluor 555 (red) and Alexa Fluor 488 (green) goat anti-rabbit secondary antibodies against an elastase primary antibody along with DAPI nuclear stain. **B**) qRT-PCR was performed on RNA from the control and elastase shRNA treated cells. A Student's *t*-test was used to determine the significance of the down-regulation (**P < 0.01). **C **and **D**) Cells of each clone were counted every day for five days. Three independent growth curves were generated in this manner and the average of each cell count was graphed. Error bars indicate standard error and differences between parental and elastase shRNA cells were detected using a Student's *t*-test (*P < 0.05, ***P < 0.001). **E **and **F**) Cells of each clone were subjected to clonogenic assays, the resulting clones were enumerated and subjected to Student's *t-*test (**P < 0.01, ***P < 0.001).

Higher amounts of neutrophil elastase in breast cancer tissues from patients are associated with a poor prognosis [4,22]. To determine the effects of silencing elastase in breast cancer cells, MDA-MB-231 cells were treated with shRNA against elastase. Two cell clones were selected that had been treated with shRNA specific to elastase (231-Elastase1 and 231-Elastase2), or with nonspecific shRNA constructs as controls (231-Control1 and 231-Control2). Using confocal microscopy, strong expression of elastase was observed in MDA-MB-231 cells without shRNA treatment and in the control clones (Figure 1A). However, the clones treated with shRNA against elastase had reduced elastase expression (Figure 1A). qRT-PCR was performed on the clones to confirm and quantify the extent of down-regulation of elastase expression after shRNA treatment and showed that expression was significantly reduced compared to the 231-Control1 cells (Figure 1B).

In response to the down-regulation of elastase, MDA-MB-231 cells had only a moderate reduction in proliferation compared to the control clones. For example, by Day 5 of a growth curve, the 231-Elastase1 clone showed only a 50% reduction in cell number compared to the 231-Control1 clone (Figure 1C, D). To gauge whether the modest reduction in proliferation induced by knocking down elastase could decrease cell colony formation, clonogenic assays were performed. Decreased elastase expression resulted in a significantly reduced ability of MDA-MB-231 cells to form colonies compared to untreated or control-shRNA-treated MDA-MB-231 cells (Figure 1E, F).

### Elastase inhibition inhibits matrix invasion by breast cancer cells

**Figure 2:**
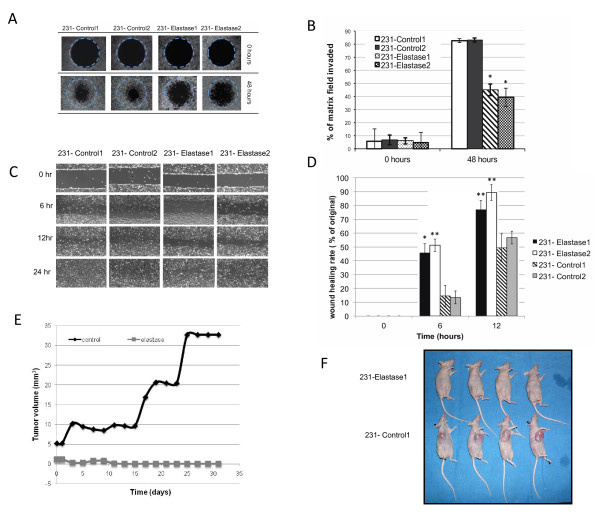
**Elastase inhibition reduces tumor cell invasion and growth of xenografts**. **A**) MDA-MB-231 cells stably expressing control, scrambled shRNA (231-Control1 and 231-Control2) or elastase-specific shRNA (231-Elastase1 and 231-Elastase2) were assessed for invasion of a collagen matrix at baseline and after 48 h. **B**) The percentage of the collagen matrix field that was invaded by each clone was quantified for triplicate wells and the mean was graphed. Significance was measured using a Student's *t*-test (*P < 0.01). **C**) Cells were also subjected to a scratch assay to detect the percentage of a scratched field that would be replaced by migrating cells within 12 hours. **D**) Three different replicates of scratch assay were performed and the average was calculated along with the standard error (shown as bars) and the *P*-value was calculated using a Student's *t*-test (*P < 0.05 compared to 231- Control 2,**P < 0.01 compared to both control clones). **E **and **F**) Cells transfected with nonspecific shRNA (control) or elastase-specific shRNA were injected into the mammary fat pad of mice, and tumor formation was observed and measured for five weeks. The results show the average of two replicate experiments.

Elastase is known to be secreted by cancer cells to invade extracellular matrix and facilitate cell migration [23]. To determine whether invasion of breast cancer cells could be abrogated by depletion of elastase, we performed an invasion assay to measure the ability of breast cancer cells to invade a collagen matrix. Results revealed that following elastase down-regulation, MDA-MB-231 could no longer invade the collagen field compared to the control cells. Specifically, in the clones with elastase knocked down, the invading cells consumed only 41% of the collagen matrix field, compared to 82% consumed by the control cells (Figure 2A, B). A scratch assay was also performed on the same cell lines to corroborate these data. After 12 hours, 77% and 89% of the scratch made in the cells with reduced elafin remained compared to 49% and 57% in the control cells (Figure 2C, D). Collectively, these data suggest that inhibition of elastase in breast cancer cells limits their invasive and migratory properties.

### Elastase facilitates tumor progression in mice

Our data, thus far, suggest that elastase affects both the proliferation and invasion of cancer cells. Therefore, we hypothesized that suppression of elastase would significantly decrease tumor burden in a xenograft model. To test this hypothesis, we injected MDA-MB-231cells transfected with control or elastase shRNA into the mammary fat pads of nude mice to form xenografts. The mice were assessed for tumor formation and tumor size daily for a month. The mice injected with breast cancer cells transfected with control shRNA developed tumors that necessitated sacrifice by 31 days; however, the mice injected with breast cancer cells transfected with elastase shRNA had minimal, mostly nonpalpable tumors for the duration (that is, 30 days) of the study (Figure 2E, F). These data suggested that elastase inhibition is sufficient for inhibition of tumor progression.

### Elastase and elafin have an inverse pattern of expression

**Figure 3:**
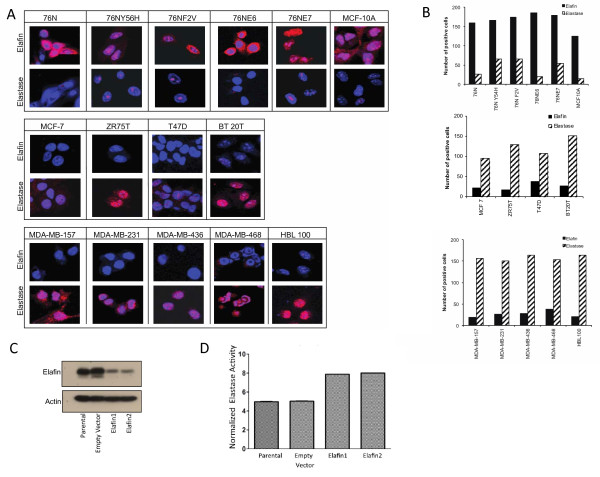
**Elafin and elastase have an inverse pattern of localization**. **A**) Non-tumorigenic breast epithelial cell lines (top panel), ER-positive breast cancer cell lines (middle panel) and ER-negative breast cancer cell lines (bottom panel) were subjected to confocal microscopy to detect the levels of elafin and elastase expression (red) and their localization in the cytoplasm and nucleus (blue). **B**) The number of cells expressing elafin and elastase for each cell line was quantified. **C) **Western blots confirm down-regulation of elafin by shRNA constructs, Elafin 1 (lane 3) and Elafin 2 (lane 4), compared to parental and empty vector control cells (lanes 1 and 2, respectively). **D**) Elastase activity was determined by measuring the amount of substrate cleaved by elastase and was detected by spectrophotometry in 76NE6 and 76NF2V clones with either an empty vector or shRNA against elafin, Elafin 1 and Elafin 2 (*P < 0.05,**P < 0.001).

Our data (Figure 2E, F) suggest that elastase inhibition could delay breast cancer progression. However, to date, there are no clinically available small molecule inhibitors of neutrophil elastase. We hypothesized that elafin, an endogenous inhibitor of elastase, inhibits elastase and that cells expressing elafin would be phenotypically similar to cells described above that lacked elastase. We initially evaluated the cellular location and level of expression of elafin and elastase in non-tumorigenic and breast carcinoma cells using confocal immunofluorescence microscopy to determine if these molecules are co-localized inside the cell (Figure 3A). The non-tumorigenic mammary epithelial cells (Figure 3A, top panel) demonstrated high levels of elafin expression within the nucleus and lower levels of elafin expression within the cytoplasm. All of these cells, except 76N, demonstrated low but detectable levels of elastase expression within the nucleus, suggesting an inverse relationship between the two proteins. In contrast, the breast carcinoma cell lines (Figure 3A, middle and bottom panels) showed overall low levels of elafin expression and high levels of elastase expression within both the nucleus and the cytoplasm. Quantification confirmed that non-tumorigenic mammary epithelial cells had high elafin expression and low elastase expression and that breast carcinoma cells had low or no elafin expression and high elastase expression (Figure 3B). These data showed that elafin, when present, may inhibit elastase seeing that elastase levels are increased in the absence of elafin.

To confirm a direct and inverse relationship between elafin and elastase, 76NE6 cells, which are non-tumorigenic and have high levels of elafin, were treated with shRNA constructs against elafin to generate two clones (Elafin 1 and Elafin 2) of cells that lacked elafin expression (Figure 3C). Decreased elafin expression in this non-tumorigenic cell line led to a significant increase in elastase activity compared to the empty vector controls suggesting a cause and effect relationship between elafin and elastase (Figure 3D).

### Adenoviral-mediated elafin expression results in growth delay in breast cancer cells

**Figure 4:**
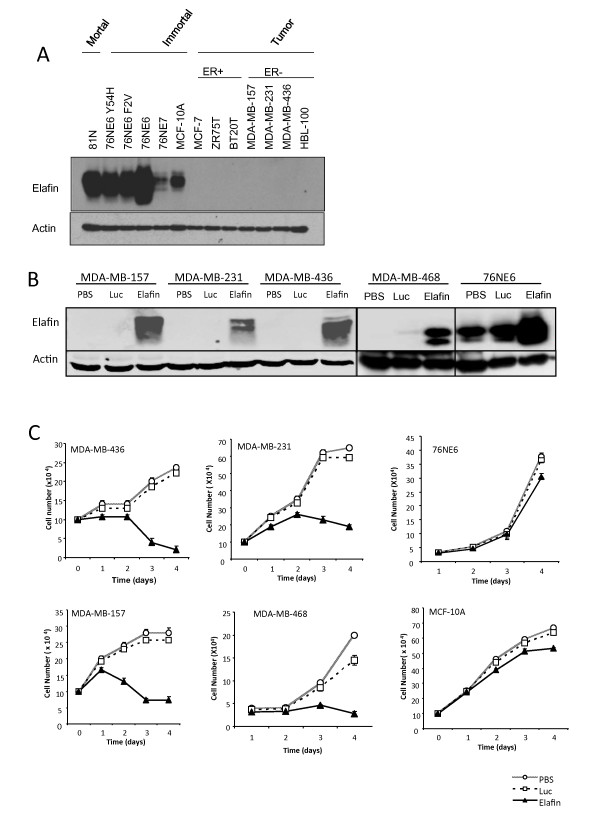
**Elafin is expressed in non-tumorigenic mammary epithelial cells but not breast cancer cells**. **A**) Lysates were collected from a panel of non-tumorigenic breast epithelial cells (mortal and immortal) and breast cancer cells (ER-positive and ER-negative) and subjected to Western blot analysis to detect elafin expression. **B**) Elafin levels were also assessed after treatment with PBS, Adenoviral luciferase (Luc), or elafin. Actin expression was assessed as a loading control. C) Breast cancer cell lines (MDA-MB-436, MDA-MB-157, MDA-MB-231 and MDA-MB-468) and non-tumorigenic breast epithelial cells (76NE6 and MCF-10A) were treated with PBS, luciferase (Luc) or elafin, cultured and counted for each day for four days.

Elafin expression differs at the level of transcription between normal mammary epithelial cells and breast carcinoma cells [7,24]. Our data (Figure 3) suggested that tumor cells lack expression of the elafin protein and that a decrease in elafin is associated with increased elastase expression and activity. To further investigate whether the differences between normal and tumor cells persist after translation, we evaluated elafin protein expression in mammary epithelial and breast carcinoma cells. Elafin protein was expressed in all of the non-tumorigenic breast epithelial cells, mortal or immortal (Figure 4A). In contrast, elafin protein was not expressed in any of the breast carcinoma cell lines, whether positive for ER or not (Figure 4A), suggesting that elafin is differentially expressed at the protein level between normal and tumor cells.

The dynamic relationship between elafin and elastase and the observation that elafin is mainly expressed in normal cells and not detectable in tumor cells (Figure 4A) led us to hypothesize that breast cancer cells expressing elafin have decreased tumorigenic potential, similar to that observed in breast cancer cells with elastase inhibited by shRNA (Figures 1, 2, 3). To test this hypothesis, we evaluated the impact of elafin expression on cell growth and viability. The 76NE6 cells with high endogenous elafin expression (Figure 4A), and four breast carcinoma cell lines with low elafin expression were infected with a recombinant adenovirus containing the luciferase reporter gene (Ad-Luc) or the elafin transgene (Ad-Elafin). The 76NE6 cells had markedly increased elafin expression after infection with Ad-Elafin (Figure 4B). The breast carcinoma cell lines, which had low endogenous elafin expression (Figure 4A) upon infection with Ad-Elafin, expressed elafin at similar levels to what is detected at baseline in the normal mammary epithelial cells (Figure 4B).

As shown in Figure 4C, in the non-tumorigenic mammary epithelial cells (76NE6 and MCF-10A), there was no demonstrable decrease in cell growth following treatment with PBS, Ad-Luc or Ad-Elafin, despite the high levels of elafin overexpression achieved (Figure 4B). In contrast, in each of the breast carcinoma cell lines expressing elafin (via Ad-elafin) at the physiological levels of what is found in normal cells, there was a reduction in cell number over time (Figure 4C). As expected, there was no substantial difference in cell growth between breast carcinoma cells treated with Ad-Luc and those treated with PBS, showing that the inhibition was attributable to the presence of elafin. There was significant apoptotic cell death in the Ad-Elafin-treated breast carcinoma cells compared to the Ad-Luc-treated breast carcinoma cells (*P *< .001; Additional file 2). Therefore, elafin expression negatively regulates the proliferation of breast cancer cells in part through induction of apoptosis.

### Elafin treatment results in growth delay of established xenografts

**Figure 5:**
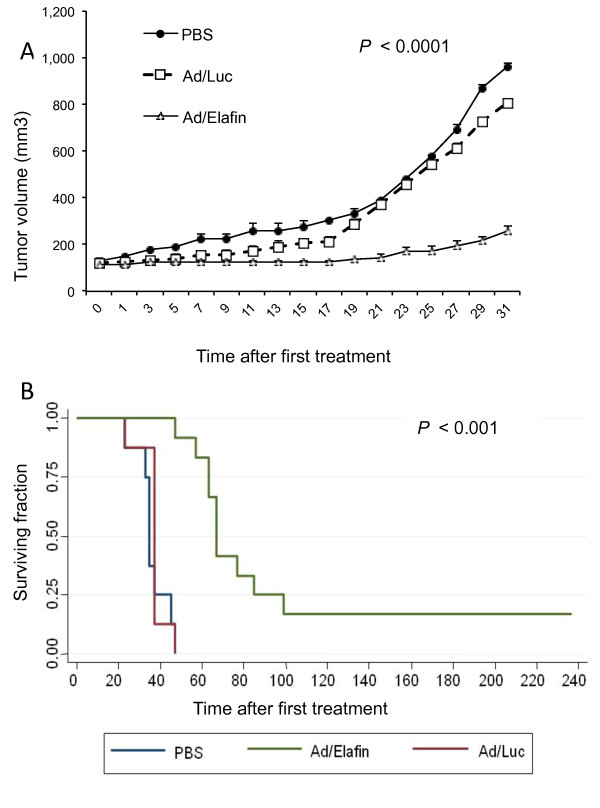
**Elafin treatment of mice with breast cancer xenografts leads to increased survival**. Nude mice with established MDA-MB-468 xenografts were treated with Ad-Elafin (*n *= 6), PBS (*n *= 4) or Ad-Luc (*n *= 4). **A**) Tumor volume was measured and recorded every other day. Mice were sacrificed when tumors were greater than 1.5 cm in diameter, and **B**) survival was recorded. The results show the average of two replicate experiments.

Elastase inhibition by shRNA provides a means to decrease the tumor burden in a xenograft model (Figure 2C, D). To further assess if overexpression of elafin and down-regulation of elastase have similar physiological endpoints, we next investigated the effect of elafin expression on tumor progression in an *in vivo *model. MDA-MB-468 cells were injected into the mammary fat pad of nude mice and were then treated with Ad-Luc, PBS or Ad-Elafin and the tumor burden was monitored over the duration of the study. Tumors in the mice treated with Ad-Luc or PBS continued to grow, requiring sacrifice within 45 days. However, there was an immediate cessation in tumor growth in the mice treated with Ad-Elafin (*P *< .0001, Figure 5A). All of the mice treated with Ad-Elafin remained alive for at least 45 days after initial treatment (Figure 5B). Ten of the 12 mice treated with Ad-Elafin experienced tumor growth necessitating sacrifice between Days 50 and 100. At eight months after initial treatment, one mouse treated with Ad-Elafin had experienced a decrease in tumor size to less than 30 mm^3^, and one had experienced complete resolution of the tumor. Elafin treatment resulted in significantly improved event-free survival compared with PBS or Ad-Luc treatment (*P *< 0.001, Figure 5B).

### Elafin loss is associated with ER-positive, poor-prognosis breast cancer and shorter time to relapse

**Figure 6:**
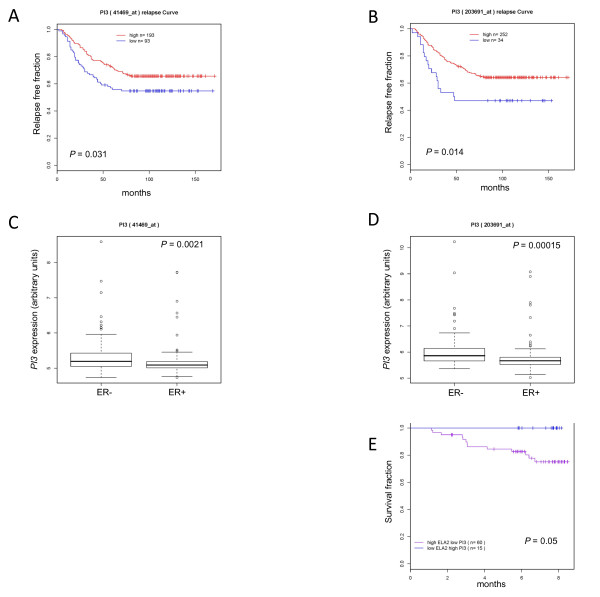
**Lower expression of the elafin gene (*PI3*) indicates a poor prognosis**. *PI3 *expression evaluated using two separate probes (A and C use 41469_at; B and D use 203691_at) was extracted from previously published datasets and correlated with **A **and **B**) time to relapse along with **C **and **D**) ER expression by using a Kaplan Mier survival analysis. **E**) Expression of the *ELA2 *gene (which encodes for elastase) in combination with the *PI3 *gene was also correlated with disease-specific survival.

We next asked if changes in expression of elafin or elastase in breast tumors are correlated with changes in patient outcome. To this end, we assessed elafin gene (*PI3*) expression in previously published microarray data from node-negative breast cancer patients [15]. On the basis of expression of *PI3*, as detected by two probes, the patients in the cohort were stratified as having high or low expression. High *PI3 *expression was associated with a longer time to relapse (*P *= .027) (Figure 6A, B). By 48 months, 63% of patients with low levels of elafin had had a relapse. In contrast, by 80 months, 64% of patients with high levels of elafin remained free of disease. Additionally, lower levels of elafin expression were associated with ER-positive tumors (*P *= .00015, Figure 6C, D). These data suggest that loss of elafin correlates with a subset of breast cancers and may contribute to their distinct phenotype. Overall, the data from the Wang *et al. *cohort suggested that low elafin expression is an indicator of poor prognosis in patients with lymph node-negative breast cancer.

Analysis of a second microarray dataset [25] supported these findings and showed that patients with the combination of high levels of elastase (*ELA2*) expression concomitant with low levels of elafin expression were more likely to relapse and die from their breast cancer sooner after diagnosis than patients with high elafin expression and low elastase expression (*P *= .05, Figure 6E). After eight months, the proportion of patients alive was more than 20% higher in the elafin-high, elastase-low group. These data showed that elafin and elastase have an inverse relationship and that increased elastase expression and decreased elafin expression correlate with a poor prognosis in breast cancer patients.

## Discussion

In this report, we show an inverse relationship between elastase and elafin protein expression and physiological functions in cell lines, in mice and in patients. In non-tumorigenic cell lines, elafin was detected, but elastase levels were low. In tumor cell lines, the reverse relationship was observed. To determine how an imbalance of elastase and elafin in tumor cells could increase their tumorigenic potential, we overexpressed elafin or knocked down elastase in tumor cells. We found that the presence of elafin or absence of elastase had very similar physiological consequences, resulting in the inhibition of proliferation and colony formation of the tumor cells. In addition, increased elafin or decreased elastase expression in mice resulted in decreased tumor size and increased their survival. Lastly, in an analysis of microarray data from breast cancer patients, the combination of high levels of elafin and low levels of elastase was associated with longer time to relapse. These results suggest a very tight cross talk between elafin and elastase across all model systems examined.

It is reasonable to infer from our findings that a downward shift in elafin or an upward shift in elastase could provide a tumor with the environment needed to grow and progress. The pathways that this machinery activates are likely both proliferation and invasion as both pathways were shown to be decreased with down-regulation of elastase. Elastase has been implicated in cleaving several substrates that play direct roles in mediating these tumor-promoting pathways. For example, elastase has been implicated in the cleavage of cyclin E into its low-molecular-weight forms, which are capable of deregulating the cell cycle [26-28], and this cleavage is inhibited by elafin (data not shown). We have shown in this work (Figure 6B) that exogenous elafin expression in tumor cells induces apoptosis to result in tumor suppression. This confirms previous data showing elafin dose dependent mediated apoptosis in breast cancer cells that lacked pRb, but had a functional caspase-3 [29]. Others have shown that elafin mediates apoptosis through a p53-dependent pathway in melanoma cells [30]. Elafin has also been shown to induce apoptosis by inhibiting elastase-mediated cleavage of CD14 [31]. Elastase is implicated in the cleavage of cut homeobox 1 which accelerates S-phase entry and is inversely correlated with survival [32-34]. Further research will be needed to elucidate the pathways regulated by the elastase/elafin switch.

Gene array data have previously been used to identify gene signatures associated with breast cancer subtypes and are invaluable tools for identifying genes associated with disease outcome. We mined published datasets to analyze the elafin gene expression in relation to time to relapse. The combination of high elafin and low elastase was associated with longer time to relapse. Because elafin is regulated at the level of transcription, it will be necessary to analyze elafin expression at the protein level to further investigate its role in the various breast cancer subtypes. The signal for elastase gene expression was relatively low, which supports previous reports that neutrophils are a source of elastase and that it is taken up in an active form by the cancer cells via endocytosis [35,36].

Manipulating the reciprocal relationship between elastase and elafin to increase elafin expression could prove beneficial to breast cancer patients. In combination with targeted treatments or chemotherapy, inhibition of elastase would inhibit tumor promoting activity in both the tumor cell and tumor environment. The efficacy of treatment strategies designed to increase elafin expression could be predicted by the presence of elastase substrates, such as low-molecular-weight cyclin E. Because elafin is an endogenously expressed human protein, it could serve as the ideal candidate for inhibiting elastase. Furthermore, these data provide a rationale for testing elafin as a prognostic marker in a prospective study.

## Conclusions

In this study we show that elafin and elastase have a reciprocal, but co-localized pattern of expression. Normal cells express higher amounts of elafin and low levels of elastase expression whereas tumor cells have higher elastase expression and minimal levels of elafin. Overexpression of elafin reduced proliferation of tumor, but not normal, cell lines and growth of tumor cell xenografts. Additionally, silencing elafin increased elastase activity. Because of the role elafin plays in inhibiting elastase and reducing breast cancer cell proliferation, we hypothesized that it could be used as a prognostic marker in breast cancer patients. Using microarray data, we showed the low elafin expression is correlated with poor outcome. Therefore, expression of elafin is an ideal candidate for a therapeutic inhibition of elastase mediated breast cancer progression and as a prognostic marker for breast cancer.

## Abbreviations

Ad-Elafin: Adenoviral vector with elafin transgene; Ad-Luc: Adenoviral vector with luciferase transgene; DAPI: 4',6-diamidino-2-phenylindole; E1: Adenovirus early region 1; E1A: Adenovirus early region 1A; E3: Adenovirus early region 3; ER: Estrogen receptor; PBS: Phosphate-buffered saline; SDS-PAGE: Sodium dodecyl sulfate polyacrylamide gel electrophoresis; shRNA: Short hairpin RNA; vp: Viral particles.

## Competing interests

The authors of this paper declare that they have no competing interests.

## Authors' contributions

KK and KKH conceived of the study, supervised its design and coordination, and drafted the manuscript. YL performed all experiments and analyses except where noted below. TY performed experiments for Figure 4A, B and participated in the study design. MY and FZ performed data mining and statistical analysis for Figure 6. C-HS carried out the experiments for Figure 3C. HW helped analyze and interpret the data and revise the manuscript. GBM, FB and MZ contributed to the concept and design of the study and critically revised the paper. All authors read and approved the final manuscript.

## Supplementary Material

Additional file 1 **Uncropped Western blots**. Western blots for actin and elafin are shown before they were cropped for Figures 3 and 4. Click here for file

Additional file 2 **Ad-Elafin induces apoptosis in breast cancer cells, but not normal cells**. To determine whether apoptosis contributed to the decreased cell proliferation and colony formation observed in the Ad-Elafin-treated breast carcinoma cells, breast cancer cells (MDA-MB-436) and non-tumorigenic mammary epithelial cells (76NE6) were transfected with Ad-Luc, transfected with Ad-Elafin, or treated with PBS and stained with propidium iodide, and the apoptotic fraction was determined by flow cytometry. There was significant apoptotic cell death in the Ad-Elafin-treated breast carcinoma cells compared to the Ad-Luc-treated breast carcinoma cells (*P *< .001). There was no significant apoptosis noted in the normal mammary epithelial cells treated with Ad-Elafin, demonstrating that elafin induces apoptosis in cancer cells, causing tumor-specific growth inhibition. These data showed that elafin expression negatively regulates the proliferation of breast cancer cells at least partially through induction of apoptosis. Analysis of apoptosis was performed with propidium iodide staining. All cells (floating and adherent) were centrifuged at 2,000 rpm for five minutes at 4°C. The cell pellets were then washed once with PBS and resuspended with 1 μl of propidium iodide per sample. The sub-G_1 _fraction was analyzed using a FACS Calibur flow cytometer (BD Biosciences, San Jose, CA, USA). Click here for file
