# Supplementary material for: Elafin, an inhibitor of elastase, is a prognostic indicator in breast cancer
Source: Breast Cancer Res. 2013 Jan 15;15(1):R3. doi: 10.1186/bcr3374 (PMC3672770; doi:10.1186/bcr3374)
Supplement: Additional file 1 — Uncropped Western blots. Western blots for actin and elafin are shown before they were cropped for Figures 3 and 4. [file bcr3374-S1.DOCX]

Additional file 1:
