# Supplementary figures and images for: Elafin, an inhibitor of elastase, is a prognostic indicator in breast cancer
Source: Breast Cancer Res. 2013 Jan 15;15(1):R3. doi: 10.1186/bcr3374 (PMC3672770; doi:10.1186/bcr3374)

**Additional File 2:**


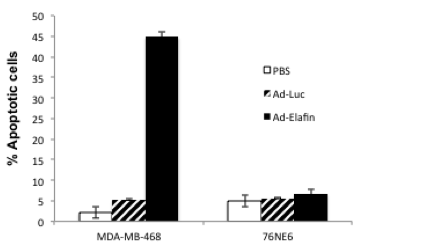

Supplement: Additional file 2 — Ad-Elafin induces apoptosis in breast cancer cells, but not normal cells. To determine whether apoptosis contributed to the decreased cell proliferation and colony formation observed in the Ad-Elafin-treated breast carcinoma cells, breast cancer cells (MDA-MB-436) and non-tumorigenic mammary epithelial cells (76NE6) were transfected with Ad-Luc, transfected with Ad-Elafin, or treated with PBS and stained with propidium iodide, and the apoptotic fraction was determined by flow cytometry. There was significant apoptotic cell death in the Ad-Elafin-treated breast carcinoma cells compared to the Ad-Luc-treated breast carcinoma cells (P < .001). There was no significant apoptosis noted in the normal mammary epithelial cells treated with Ad-Elafin, demonstrating that elafin induces apoptosis in cancer cells, causing tumor-specific growth inhibition. These data showed that elafin expression negatively regulates the proliferation of breast cancer cells at least partially through induction of apoptosis. Analysis of apoptosis was performed with propidium iodide staining. All cells (floating and adherent) were centrifuged at 2,000 rpm for five minutes at 4°C. The cell pellets were then washed once with PBS and resuspended with 1 μl of propidium iodide per sample. The sub-G1 fraction was analyzed using a FACS Calibur flow cytometer (BD Biosciences, San Jose, CA, USA). [file bcr3374-S2.DOCX]
